# Time series of useful energy consumption patterns for energy system modeling

**DOI:** 10.1038/s41597-021-00907-w

**Published:** 2021-05-31

**Authors:** Jan Priesmann, Lars Nolting, Christina Kockel, Aaron Praktiknjo

**Affiliations:** 1 RWTH Aachen University, Institute for Future Energy Consumer Needs and Behavior (FCN), Chair for Energy System Economics (FCN-ESE), Mathieustr. 10, 52074 Aachen, Germany; 2 JARA-ENERGY, 52074 Aachen, Germany

**Keywords:** Energy supply and demand, Energy economics, Energy modelling, Energy efficiency

## Abstract

The analysis of energy scenarios for future energy systems requires appropriate data. However, while more or less detailed data on energy production is often available, appropriate data on energy consumption is often scarce. In our JERICHO-E-usage dataset, we provide comprehensive data on useful energy consumption patterns for heat, cold, mechanical energy, information and communication, and light in high spatial and temporal resolution. Furthermore, we distinguish between residential, industrial, commerce, and mobility consumers. For our dataset, we aggregate bottom-up data and disaggregate top-down data both to the NUTS2 level. The NUTS2 level serves as an interface to validate our combined method approach and the calculations. We combine a multitude of data sources such as weather time series, standard load profiles, census data, movement data, and employment figures to increase the scope, validity, and reproducibility for energy system modeling. The focus of our JERICHO-E-usage dataset on useful energy consumption might be of particular interest to researchers who analyze energy scenarios where renewable electricity is largely substituted for fossil fuel (sector coupling).

## Abstract (summary)

**Table:** 

Measurement(s)	space heat consumption patterns • hot water consumption patterns • space cooling consumption patterns • process cooling consumption patterns • mechanical energy consumption patterns • information and communication (ICT) consumption patterns • light consumption patterns • mileage profiles • process heat consumption patterns
Technology Type(s)	computational modeling technique
Factor Type(s)	final energy consumption pattern • final energy annual consumption • heating system dimensioning guidelines • census data • traffic census data • weather data • economic reports and statistics • technical reports and statistics
Sample Characteristic - Environment	spatiotemporal region • temperature of air • building • economy • industry • demography • energy

Machine-accessible metadata file describing the reported data: 10.6084/m9.figshare.14039456

## Background & Summary

To successfully mitigate greenhouse gas emissions, energy systems must be transformed through global endeavors. Open data can help drive and support these efforts^1,2^. This does not only apply to the generation side, which is changing from centralized fossil to decentralized renewable energy conversion. It also concerns energy consumption, which is transforming (1) through energy efficiency measures and (2) the change in end-user technologies (e.g. the adoption of heat pumps or battery electric vehicles). Ultimately, the transformations on the generation and consumption sides define the infrastructure requirements for the distribution of energy carriers^3^.

**Fig. 1:**
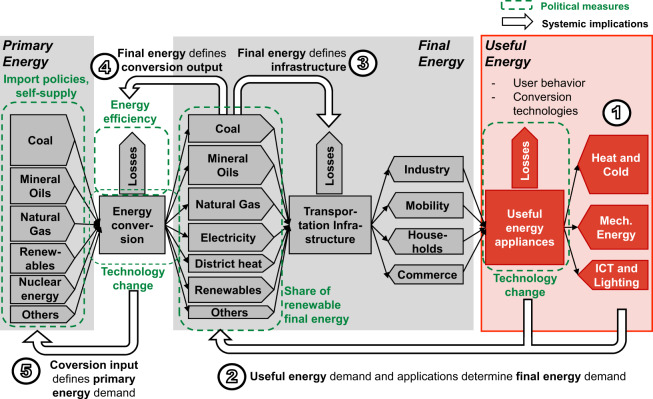
Energy balance of a national energy system based on Zweifel, Praktiknjo, and Erdmann^36^.

Energy system modeling is becoming increasingly important for the analysis of energy scenarios and their implications on infrastructure and society. However, a thorough analysis of energy systems requires appropriate data. A widespread source for such data are energy balances. Energy balances comprise data on the procurement, conversion, and consumption of energy carriers. Figure 1 shows an exemplary energy balance. Energy balances are usually divided into primary and final energy, energy conversion, and energy consumption within the residential, industrial, commerce, and mobility sectors. Despite being valuable for energy system analysis, energy balances lack multiple information that would be highly valuable to analyze future energy systems:

1. Energy balances do not comprise the last step of energy conversion which consumers take using their end-use appliances (e.g., heating systems, cars, light bulbs) to convert final energy (e.g., electricity, natural gas, gasoline, diesel) into the eventually sought-after useful energy (e.g., heat, mechanical energy, light) at the end-consumer level. While some reports name the different types of useful energy categories that the final energy carriers can be associated with, these reports do not include actual quantitative data on useful energy.
2. Energy balances are usually not available on a sub-national level (in Germany, energy balances are available on the federal level but in varying quality and depth of detail).
3. Energy balances report annual energy quantities and are therefore not available in a temporal resolution of less than one year.

Future renewable energy systems are likely to be centered around renewable electricity as the main final energy carrier that will be converted into the different types of useful energy using appliances such as heat pumps or electric vehicles or via indirect electrification based on, e.g., hydrogen^4^. With the increasing share of volatile renewable electricity generation and changing consumption patterns, the temporal distribution of energy consumption becomes increasingly important for the outline of future energy systems. At the same time, the spatial distribution of energy consumption gains importance as the production of renewable electricity and its consumption regionally decouple.

**Fig. 2:**
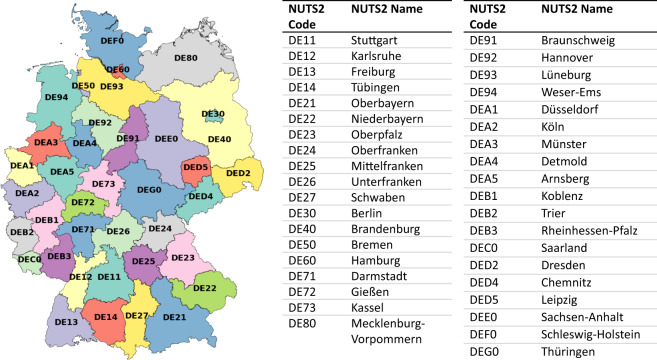
38 NUTS2 regions included in the dataset.

Our JERICHO-E-usage dataset is an extension of the national energy balance that depicts the so far unattended consumption of useful energy in high spatio-temporal resolution. We use 2019 as our base year. As we publish our source code, the JERICHO-E-usage dataset can be updated in the future. Further, the geographical scope can be extended by applying the described methods to other countries. The dataset is spatially resolved at the NUTS2 level comprising 38 regions (see Fig. 2 and Table 1) and temporally resolved in hours (NUTS refers to *Nomenclature des unités territoriales statistiques* of the European Union *–* a hierarchical system in which regions are administrative levels or units). We perform our calculations separately for the residential, industrial, commerce, and mobility sectors. We use combinations of bottom-up and top-down approaches. For this, we aggregate bottom-up data and disaggregate top-down data both to the NUTS2 level. The NUTS2 level serves as an interface to validate our calculations. We combine a multitude of data sources such as weather time series, standard load profiles, census data, movement data, and employment figures, among others. Overall, our dataset increases:

1. the *scope* of possible investigations with respect to future energy systems with high degrees of sector-coupling as our dataset allows for investigations in high spatio-temporal resolution,
2. the *validity* of these analyses as they no longer rely on simplified distribution keys for consumption patterns, and
3. the *reproducibility* as we provide a common input data framework.

Our JERICHO-E-usage dataset covers consumption patterns for eight types of useful energy in the residential, industrial, and commerce sectors. For the mobility sector, we provide the consumption patterns for mechanical energy and additionally mileage profiles for six vehicle types. For more details, see the Data Records section. Our dataset can be used to outline possible sector coupling pathways and analyze their systemic implications as shown in the Usage Notes section. As discussed in the Technical Validation section, our dataset and methodology can further improve energy system analyses by featuring spatial and sectoral resolutions of energy consumption patterns that were so far not publicly available.

## Methods

We divide useful energy into the following types: consumption patterns for (1) space heating, (2) hot water, (3) process heat, (4) space cooling, (5) process cooling, (6) mechanical energy, (7) information and communication technology, and (8) lighting. The underlying application purpose of useful energy differs among the residential, the industrial, the commerce, and the mobility sectors. The following methods can be applied generically and are not limited to the German case.

**Fig. 3:**
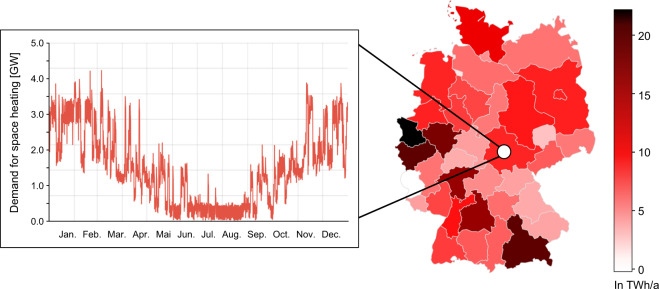
Spatio-temporal space heating consumption patterns as an illustration of the regional and temporal scopes of the dataset. The regional scope comprises 38 regions (NUTS2 level). The temporal scope comprises one year with 8760 hours.

For the combined top-down and bottom-up approach, we use the NUTS2 level as the validation interface. In our top-down model, we disaggregate the energy balances for Germany from 2019 down to the NUTS2 level for all sectors and forms of useful energy^5^. We define the bottom-up model at the NUTS3 level and aggregate the resulting data to the NUTS2 level. We identify main drivers for the top-down and bottom-up approaches based on methods used to generate the national energy balance^6,7^ and extend them with the help of scientific studies^8,9^. In particular, the heat demand depends on regionally specific climate conditions. We represent differences in climate conditions using 15 climate zones within Germany as defined by German Climate Agency^10^. For the temporal resolution, the time series calculated account for climate zone-specific temperature and occultation time series. Besides, the time series are created according to the calendar of the year 2019, i.e., distribution of weekdays, Saturdays, as well as Sundays, and public holidays. Figure 3 shows spatio-temporal space heating consumption patterns as an illustration of the regional and temporal scopes of the dataset.

### Residential sector

**Fig. 4:**
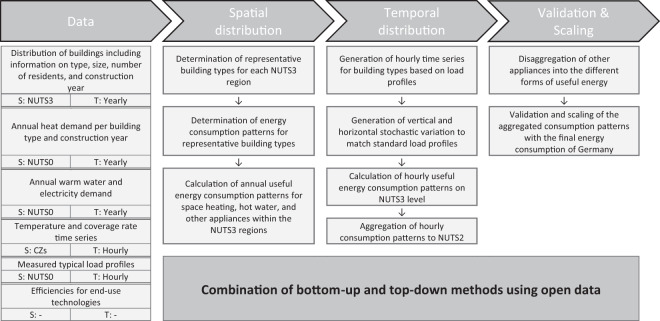
Methodology overview for the spatial and temporal disaggregation of useful energy consumption patterns in the residential sector. For each dataset, the spatial (S) and temporal (T) resolution of the original data is given.

In the residential sector, we identify the demand for heating and cooling to be the main driver for useful energy consumption patterns. We distinguish between single-family and multi-family houses and account for regional influencing factors. Figure 4 shows our methodology for the spatial and temporal distribution of the useful energy consumption for the residential sector.

In a first step, we calculate annual energy consumption (for space heating, hot water, and other appliances) per building type according to the VDI guideline 4655^11^ and data on building typologies^12^. This calculation requires data on the year of construction, the size, and the number of residents and apartments per building which we derive from census data^13^. The spatial distribution is determined by the distribution of the different residential building types in the respective NUTS3 regions according to the latest census data^13^. For each region, we define representative buildings (separated by single and multi-family houses) that are determined by the age structure of the building stock, average living areas, and regional temperature profiles. Based on this distribution, we allocate average representative consumption patterns to the regions. The annual heating consumption is scaled in correspondence to the respective climate zone.

The temporal distribution is based on measured load profiles of different types of residential buildings in hourly resolution^11^. For this, we distribute energy consumption based on a typical-day approach for the year 2019. For the consideration of simultaneity effects and user behavior, the individual time series are stochastically shifted horizontally (discrete distribution with an expected value of zero hours and a standard deviation of one hour) and vertically (normal distribution with the original value as expected value and a standard deviation of 5%) before being aggregated to representative profiles per region.

Finally, we disaggregate the regional consumption time series for other appliances into different useful energy categories. We transform the time series to relative time series in reference to the total consumption across all regions. We then calculate the total consumption for useful energy for all categories of useful energy except space heating and hot water, as these time series were already calculated in the first step. We multiply the relative time series by the total consumption of useful energy.

**Fig. 5:**
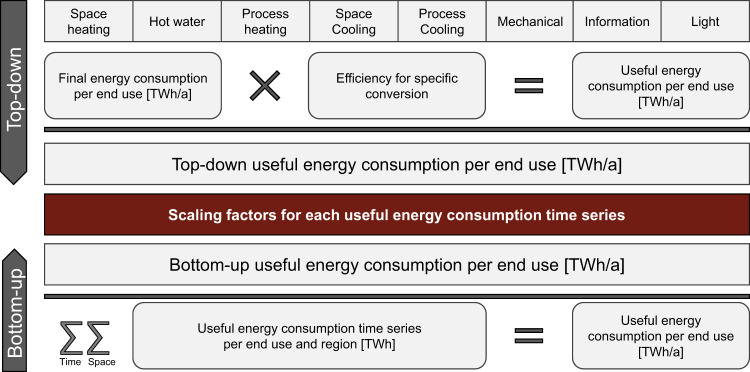
Methodology applied for validating and scaling the spatio-temporal time series of useful energy consumption.

Figure 5 shows the validation and scaling procedure for the residential sector. In the top-down approach, we use the aggregated data for final energy^5^ and data on conversion efficiencies^14^ to calculate total annual useful energy consumption per end-use. In the bottom-up approach, we aggregate the useful energy consumption per end-use across all regions and hours. By comparing the resulting total annual useful energy consumption, we derive scaling factors that we apply to our spatio-temporal time series.

### Industrial and commerce sectors

**Fig. 6:**
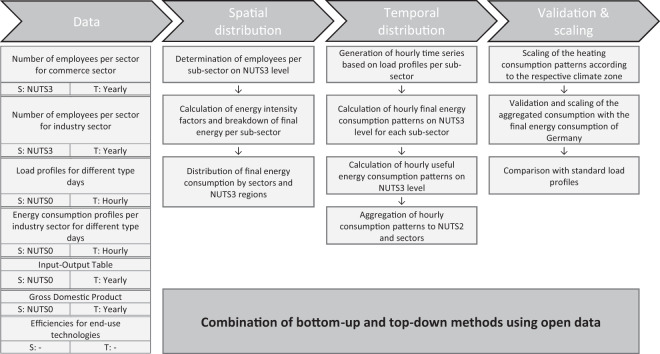
Methodology overview for the spatial and temporal disaggregation of the useful energy consumption for the industrial and the commerce sectors. For each dataset, the spatial (S) and temporal (T) resolution of the original data is given.

For the industrial and commerce sectors, we calculate the useful energy consumption patterns by combining top-down and bottom-up models as outlined in Fig. 6.

Starting with the annual final energy consumption for the German commerce and industrial sectors^5^, we then disaggregate the data spatially to the NUTS3 level based on sub-sector-specific employment figures and energy intensities within these sub-sectors. The sector-specific energy intensities correspond to the energy-weighted added value that we calculate based on the input-output table for Germany of the year 2015^15^ (see Table 1). The sub-sectors comprise agriculture, trade, public, and finance for the commerce sector and food industry, manufacture of glassware and ceramics, car industry, mechanical engineering, chemical industry, paper industry, metal production and processing, and other industry for the industrial sector. We extracted the number of employees in each NUTS3 region for the commerce sector from official data by the statistical office of the federal states^16^ and the industrial sector from the national^17^ and European^18^ statistical offices. We manually filled missing data points based on online research on the respective companies at locations with insufficiently accurate data.

**Table 1:** Overview of energy intensity factors and distribution of useful energy consumption for the different sub-sectors^5,15^.

Sector	Sub-sectors	Factor for energy intensity (normalized)	Distribution of the total useful energy consumption (in %)
**Industry**	Food industry	0.06	4.20
	Manufacturer of glassware and ceramics	0.20	5.60
	Car industry	0.03	7.00
	Mechanical engineering	0.04	9.70
	Chemical industry	0.17	19.10
	Paper industry	0.19	5.90
	Metal production and processing	0.21	26.60
	Others	0.10	21.90
	**Sum**	**1.00**	**100.00**
**Commerce**	Agriculture	0.31	22.50
	Trading	0.22	15.30
	Financial	0.22	23.60
	Public	0.25	38.60

Furthermore, the temporal disaggregation is based on a bottom-up approach, in which representative time series for each sub-sector in each NUTS3 region are determined based on typical days for the different industrial^19^ and the commerce^20^ sub-sectors. We translate the time series of final energy consumption to time series of useful energy consumption using end-use-specific conversion efficiencies^14^. Finally, the data is aggregated to the NUTS2 level.

### Mobility sector

**Fig. 7:**
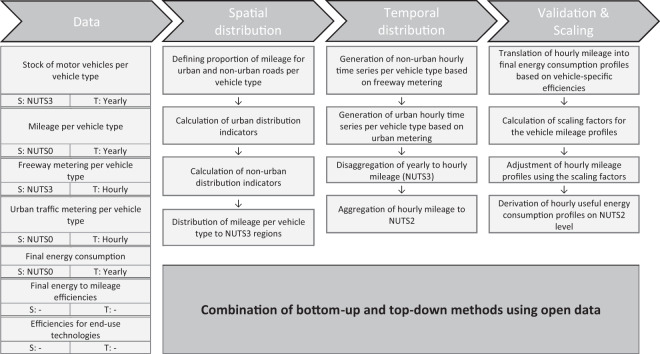
Methodology overview for the spatial and temporal disaggregation of the consumption in the mobility sector. For each dataset, the spatial (S) and temporal (T) resolution of the original data is given.

We estimate the consumption of useful energy in the mobility sector based on the regional driving profiles of different vehicles and road types. Figure 7 shows our methodology for the spatial and temporal distribution of the consumption in the mobility sector in Germany’s NUTS2 regions.

The spatial distribution of vehicle mileage depends on (1) the types of vehicles, (2) the average annual mileage per vehicle type, and (3) the mileage shares of urban and non-urban roads per vehicle type.

Table 2 gives an overview of these indicators. The vehicles and mileages are spatially distributed to the NUTS3 regionals using official vehicle stock and mileage statistics^21,22^. We then temporally disaggregate the yearly values to an hourly resolution. For this, we generate vehicle-specific time series for urban^23^ and non-urban^24^ mileage and assign mileage shares for the two road types to each of the vehicle types. We differentiate between the final energy sources diesel, gasoline, gas, electricity, and biofuels and account for engine sizes and other vehicle-specific features by using individual conversion efficiencies per vehicle type. After having calculated the hourly mileage for NUTS3 regions, we again aggregate the data to the NUTS2 level.

**Table 2:** Overview of the differentiated vehicle types and their average mileage and refueling proportions. Average mileage per year is taken from KBA^22^.

Vehicle type	Average mileage per year	Mileage shares (urban/non-urban)
Passenger car	13,922	75%/25%
Motorcycle	2,250	75%/25%
Bus	57,580	82%/18%
Truck (less than 3.5 t)	19,371	75%/25%
Truck (more than 3.5 t)	21,655	0%/100%
Semitrailer	96,915	0%/100%
Other vehicles	9,189	0%/100%

To validate and scale the mileage profiles, we use the annual final energy consumptions of diesel, gasoline, gas, biomass, and electricity in the road traffic sector^25^. The mileage profiles for the different vehicle types influence multiple final energy consumption – e.g., the mileage profiles for passenger cars influence the consumption of diesel, gasoline, gas, biomass, and electricity. To solve the resulting system of equations, we set up a linear programming (LP) problem as follows:

$$\mathop{\min }\limits_{{\alpha }_{v,}{\beta }_{v,f}^{FE},{\gamma }_{f}^{FE},{\delta }_{f}^{FE,pos},{\delta }_{f}^{FE,neg}}\sum _{f\in F}({\delta }_{f}^{FE,pos}+{\delta }_{f}^{FE,neg})$$

$$\begin{array}{cc}{s.t.}\quad {\alpha }_{v}\cdot {{\rm{A}}}_{v,f}^{Fuel\;Share}\cdot {{\rm{B}}}_{v,f}^{Conversion}\cdot \sum _{n\in N}\sum _{h\in H}{\Gamma }_{v,n,h}^{Mileage}={\beta }_{v,f}^{FE} & \forall v\in V,f\in F\\ \sum _{v\in V}{\beta }_{v,f}^{FE}={\gamma }_{f}^{FE} & \forall f\in F\\ {\gamma }_{f}^{FE}-{{\rm{E}}}_{f}^{AGEB}={\delta }_{f}^{FE,pos}-{\delta }_{f}^{FE,neg} & \forall f\in F\\ {\alpha }_{v}{\ge \Delta }^{Lowerlimit} & \forall v\in V\\ {\alpha }_{v}{\le \Delta }^{Upperlimit} & \forall v\in V\\ {\delta }_{f}^{FE,pos},{\delta }_{f}^{FE,neg}\ge 0 & \forall f\in F\end{array}$$

Here, the objective of the LP problem is to minimize the deviation in final energy consumption ${\delta }_{f}^{FE}={\delta }_{f}^{FE,pos}+$ ${\delta }_{f}^{FE,neg}$ between the consumption stated by the national statistics ${{\rm{E}}}_{f}^{AGEB}$ and our bottom-up data ${\gamma }_{f}^{FE}$ for the relevant final energy type $f\in F$. By solving the LP problem, the factors *α*_*v*_ are defined that scale the mileage profiles for the different vehicle types $v\in V$. The contribution of each vehicle type *v* to the final energy consumption of *f* is represented by the variable ${\beta }_{v,f}^{FE}$. The parameter ${{\rm{A}}}_{\nu ,f}^{Fuel\;share}$ defines the mileage share for a vehicle type that is allocated to a specific fuel, e.g. the share of mileage that can be accounted to diesel-fueled passenger cars^22,26^. The parameter ${{\rm{B}}}_{\nu ,f}^{Conversion}$ defines the average conversion efficiency per vehicle type from final energy to mileage^27^. The mileage per vehicle type, region, and hour is represented by the parameter ${\Gamma }_{v,n,h}^{Mileage}$. We incrementally decrease and increase the parameters Δ^*Lower limit*^ and Δ^*Upper limit*^ starting from one until the total deviation ${\delta }_{f}^{FE}$ converges to zero. The results show that the mileage for trucks was underestimated in the initial profiles. This was expected as Germany is a transit country for road transportation which could not be depicted solely with the German vehicle stock.

Finally, useful energy consumption time series are calculated using the scaled mileage profiles and end-use-specific conversion efficiencies^14^. We restrict the useful energy types to mechanical energy for the mobility sector as other consumption is negligibly small. We include both, the useful energy consumption as well as the mileage per vehicle type in the JERICHO-E-usage dataset.

## Data Records

The JERICHO-E-usage dataset is available on 10.6084/m9.figshare.c.5245457^28^ and visualized in an interactive tool on https://jericho-energy.de/e-usage. All data are spatially resolved at the NUTS2 level and temporally resolved in hours. The dataset includes 28 time series of useful energy and energy service consumption per NUTS2 region (see Table 3).

**Table 3:** Overview of the 28 consumption profiles per NUTS2 region that we include in our dataset.

Sector	Useful energy type/Energy service	Description
Residential	Space heating	Space heating demand in the residential sector [kW]
	Hot water	Hot water demand in the residential sector [kW]
	Process heat	Process heat demand in the residential sector [kW]
	Space cooling	Space cooling demand in the residential sector [kW]
	Process cooling	Process cooling demand in the residential sector [kW]
	Mechanical energy	Mechanical energy demand in the residential sector [kW]
	Information and communication (ICT)	ICT demand in the residential sector [kW]
	Light	Light demand in the residential sector [kW]
Industry	Space heating	Space heating demand in the industrial sector [kW]
	Hot water	Hot water demand in the industrial sector [kW]
	Process heat	Process heat demand in the industrial sector [kW]
	Space cooling	Space cooling demand in the industrial sector [kW]
	Process cooling	Process cooling demand in the industrial sector [kW]
	Mechanical energy	Mechanical energy demand in the industrial sector [kW]
	Information and communication (ICT)	ICT demand in the industrial sector [kW]
	Light	Light demand in the industrial sector [kW]
Commerce	Space heating	Space heating demand in the commerce sector [kW]
	Hot water	Hot water demand in the commerce sector [kW]
	Process heat	Process heat demand in the commerce sector [kW]
	Space cooling	Space cooling demand in the commerce sector [kW]
	Process cooling	Process cooling demand in the commerce sector [kW]
	Mechanical energy	Mechanical energy demand in the commerce sector [kW]
	Information and communication (ICT)	ICT demand in the commerce sector [kW]
	Light	Light demand in the commerce sector [kW]
Mobility	Mechanical energy	Mechanical energy demand in the mobility sector [kW]
	Mileage passenger cars	Mileage of passenger cars [km]
	Mileage motorcycles	Mileage of motorcycles [km]
	Mileage trucks < 3.5 t	Mileage of trucks below 3.5 t [km]
	Mileage trucks > 3.5 t	Mileage of trucks over 3.5 t [km]
	Mileage semitrailer trucks	Mileage of semitrailers [km]
	Mileage other vehicles	Mileage of other vehicles [km]

We provide the data separately for the four energy sectors: Residential, industrial, commerce, and mobility. We use the classification of AGEB^5^ for the useful energy consumption that comprises space heating, hot water, process heat, space cooling, process cooling, mechanical energy, information and communication, and light. For the mobility sector, we limit the useful energy consumption to mechanical energy as the consumption of all other useful energy types is negligibly small. We additionally provide the mileage profiles in spatio-temporal resolution for the following vehicle types: Passenger cars, motorcycles, trucks below 3.5 t, trucks above 3.5 t, semitrailer trucks, and other vehicles. All energy data has the unit kW, the mileage data has the unit km.

We provide the spatio-temporal data as separate files for each sector and energy type using a single-index shape (csv, xlsx) as well as in a single file using a multi-index shape (csv, xlsx). We use the year 2019 from the final energy consumption data of AGEB^5^ as our central source for the calculation. We, therefore, adjust the timestamps of our dataset to the year 2019. The hourly values are indexed by the Central European Time (Coordinated Universal Time + 1 (UTC+1)).

## Technical Validation

As described in the Methods section, our methodology includes multiple validations and scaling steps that ensure a high validity of our dataset. In the following, we perform additional spatial and temporal validation of our dataset.

### Spatial validation

**Fig. 8:**
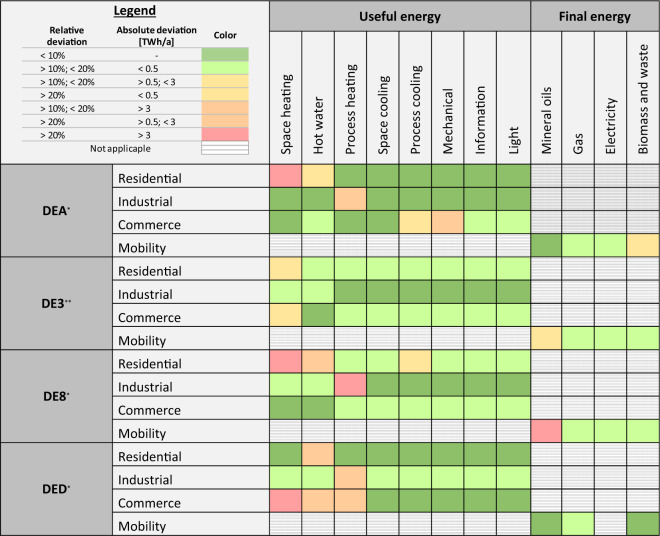
Validation of the spatial disaggregation according to regional energy balances for the federal states North Rhine-Westphalia (DEA)^37^, Berlin (DE3)^38^, Mecklenburg-Western Pomerania (DE8)^39^, and Saxony (DED)^40^.

We validate the regionalized useful energy consumption patterns against reported regional energy balances. Energy balances report on primary and final energy flows. Regional energy balances are available on the level of federal states in Germany (NUTS1). Accordingly, we aggregate our data from the NUTS2 to the NUTS1 level. For the residential, industrial, and commerce sectors, we compare the annual consumption for useful energy from our dataset to the annual consumption for useful energy that we calculate from the regional energy balances. For the mobility sector, our dataset is limited to mechanical energy. To have a more accurate validation for the mobility sector, we compare annual final energy consumption that we calculate from our dataset to annual consumption for final energy from the regional energy balances. We use data from the years 2016 and 2017, respectively, from four federal states for our validation as not all federal states report their energy balances with the required scope and at the same time. The selected federal states vary in terms of area and population density. The results of the validation are shown in Fig. 8. Numerical results for the validation can be found in the Supplementary Material.

For most regions and energy types, our dataset shows a relative deviation from the regional time series below 10% or below 20% accompanied by a small absolute deviation (marked in dark and light green). Most deviations can be found for heat consumption patterns. The deviations may stem from the time difference in the data we validate against as the most recent regional energy balances available are from 2016 and 2017.

### Temporal validation

To the best of our knowledge, there are no publicly available time series on useful energy consumption patterns on any spatial aggregation level. Therefore, we aggregate our regional time series of useful energy consumption to the NUTS0 level and translate them into final energy consumption time series. We then compare the results time series for electricity consumption to respective reported time series from entso-e^29^. The electricity consumption is reported by national transmission system operators. The reported time series are found to not fully depict the true consumption patterns as in particular industrial consumption is difficult to observe. As our consumption patterns include industrial profiles, we use further key figures to compare the peak and annual electricity consumption from entso-e^30^. The results are shown in Table 4.

**Table 4:** Validation of the temporal disaggregation according to reported time series and key figures of the electricity consumption in Germany^30,35^.

Dataset	Peak consumption [GW]	Annual consumption [TWh/a]
Hourly electricity consumption in 2019 reported by entso-e	76.3	490.5
Estimated electricity consumption for 2019 based on JERICHO-E-usage dataset	86.2	496.9
Key figures from statistical reports	84.9*	511.6

*Average measured over 35 weather years and projected for 2021. Peak consumption range from 81.6 GW to 91.2 GW.

It seems as if our dataset overestimates peak electricity consumption if only compared to the reported time series. When comparing the key figures for peak consumption we observe that our time series are close to the peak consumption reported by entso-e. The same result can be seen for annual consumption. If we account for the electricity consumption from the rail traffic of 11.5 TWh/a that is not included in our dataset the deviation to the reported annual consumption shrinks to 3.2 TWh/a. Overall, we conclude that our time series include additional information and can offer higher validity compared to aggregated reported time series due to their bottom-up nature.

## Usage Notes

### Applicability

Our JERICHO-E-usage dataset provides a starting point for appropriate data to account for energy consumption patterns in energy system analyses with high temporal and spatial resolution. For the spatial distribution of energy consumption, rather simplified distribution keys – such as the GDP per capita – are commonly used (see e.g. Robinius *et al*.^31^). Our data allows the derivation of substantially more reliable regional consumption patterns for the entire energy flows.

**Fig. 9:**
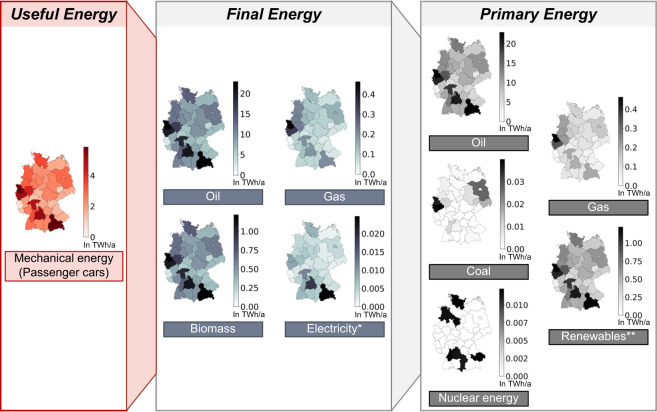
Reversed energy flow analysis. Example of the systemic implications of consumption patterns in the mobility sector in Germany assuming the current vehicle stock for the year 2019 (all values in TWh/a). *Imports are omitted in this example. Based on the electricity mix from 2019^41^. **Including biomass, wind, and solar energy. Wind and solar energy are derived using the IEA method.

Figure 9 shows an exemplary case for passenger cars. Starting with the useful energy consumption (in our example mechanical energy for the mobility of passenger cars) and by reversing the energy flow, we derive the final energy consumption depending on the end-use technology and subsequently the primary energy consumption. Our example is based on the current regional fuel distribution among passenger cars. Systemic implications of different sector coupling pathways can now easily be analyzed by e.g., increasing the share of battery electric vehicles.

With increasing degrees of sector-coupling, analyses of infrastructure requirements become more relevant causing the need for input data both in high spatial and temporal resolution. Our dataset increases the range and accuracy of possible investigations. As demonstrated in Kockel *et al*.^3^, our data can serve as input to analyze infrastructure requirements that come with higher degrees of sector-coupling.

### Limitations

To evaluate the developed disaggregation method of useful energy consumption, Germany was used as an example. Partly, because a validation basis is available (see section *Technical Validation*) and partly because it is a highly interesting use case for analyzing sector coupling scenarios with its rapidly rising share of renewable energy in the power sector while lagging in the heat and mobility sectors. However, the approach and methodology can be transferred to other regions.

Due to the novel focus on disaggregated useful energy consumption, comparable data has not been published yet. Therefore, direct verification with other data is not possible. However, the data was calculated to final energy consumption and then validated in the section *Technical Validation* with existing data. For the spatial and temporal disaggregation, generalizations had to be made. For this, the applied combination of disaggregation factors and distribution keys was each justified and validated. The largest deviations compared to the validation data were found in the residential sector, in particular for space heating consumption. This is a common problem referred to as the performance gap due to occupant behavior in buildings^32,33^.

We use a broad variety of indicators for the temporal and spatial disaggregation of macro data. However, due to partially unavailable data, we rely on some critical assumptions such as standard load profiles for the commerce sector and a spatially homogeneous mobility requirement per vehicle type. Another limitation is that the data for the mobility sector only includes road traffic. Rail, air, and water transport have been excluded from the analysis as road transport accounts for the majority of energy consumption in the mobility sector (83%).

## Supplementary information

Supplementary Material

## Data Availability

The code for compiling the time series of useful energy consumption and energy service profiles is published at https://github.com/FCN-ESE/JERICHO-E-usage^34^ under the open MIT license. Detailed instructions for using the code are included in the repository. All code is implemented in Python. For easy use of the scripts, we have added a Jupyter Notebook with further instructions on the workflow. The required input data, comprising pre-calculated data and data from official reports, are included with references.
