# Supplementary material for: Time series of useful energy consumption patterns for energy system modeling
Source: Sci Data. 2021 May 31;8:148. doi: 10.1038/s41597-021-00907-w (PMC8166825; doi:10.1038/s41597-021-00907-w)
Supplement: Supplementary file 1 — Supplementary Material [file 41597_2021_907_MOESM1_ESM.pdf]

Supplementary Material

Supplementary Table 1: Numerical values of the validation of the spatial disaggregation according to regional energy balances for the federal states North Rhine-Westphalia (DEA)[1], Berlin (DE3)[2], Mecklenburg-Western Pomerania (DE8)[3], and Saxony (DED)[4]. All values in TWh/a, the relative deviations are provided in brackets.

**[TWh/a]**		**Useful energy**								**Final energy**			
		Space heat	Hot water	Process heat	Space cooling	Process cooling	Mechanical	Information	Light	Mineral oils	Gas	Electricity	Biomass and waste
**DEA***	Residential	21.8 (37%)	3.0 (17%)	-0.9 (-1%)	-0.06 (-9%)	-1.57 (-9%)	0.01 (2%)	-0.4 (-9%)	-0.01 (9%)				
	Industrial	-1.1 (-1%)	-0.1 (-9%)	-2.2 (-14%)	0.01 (0%)	0.02 (0%)	-0.2 (0%)	0.0 (0%)	0.0 (0%)				
	Commerce	0.8 (3%)	0.3 (11%)	-0.01 (-2%)	0.2 (7%)	0.8 (12%)	1.6 (27%)	0.5 (12%)	0.08 (12%)				
	Mobility									3.4 (3%)	-0.4 (-29%)	0.1 (515%)	0.9 (16%)
**DE8****	Residential	1.3 (2%)	0.4 (22%)	0.09 (12%)	0.01 (13%)	0.2 (13%)	0.01 (18%)	0.04 (13%)	0.0 (13%)				
	Industrial	-0.08 (2%)	-0.01 (-19%)	0.1 (3%)	0.01 (8%)	0.02 (8%)	0.07 (7%)	0.0 (8%)	0.0 (8%)				
	Commerce	-0.7 (-18%)	-0.01 (-3%)	-0.1 (-14%)	0.07 (33%)	0.2 (43%)	0.1 (18%)	0.2 (43%)	0.02 (43%)				
	Mobility									-2.12 (-18%)	-0.08 (-46%)	0.01 (99%)	0.06 (13%)
**DED***	Residential	4.4 (25%)	1.1 (29%)	0.3 (19%)	0.02 (19%)	0.6 (19%)	0.04 (25%)	0.2 (19%)	0.01 19%)				
	Industrial	0.4 (32%)	0.04 (26%)	7.0 (49%)	-0.05 (-5%)	-0.08 (-5%)	-0.3 (-5%)	-0.02 (-5%)	0.0 (-5%)				
	Commerce	-0.2 (-4%)	-0.05 (-6%)	-0.3 (-18%)	0.1 (23%)	0.3 (25%)	0.3 (18%)	0.2 (25%)	0.03 (25%)				
	Mobility									4.84 (22%)	0.03 (16%)	0.04 (1262%)	0.4 (37%)
**DE3***	Residential	-0.9 (-7%)	0.7 (28%)	-0.09 (-7%)	-0.01 (-8%)	-0.2 (-8%)	-0.01 (9%)	-0.05 (-8%)	0.0 (-8%)				
	Industrial	0.2 (135%)	0.02 (118%)	3.0 (167%)	0.05 (39%)	0.09 (39%)	0.4 (4%)	0.02 (39%)	0.0 (39%)				
	Commerce	-4.1 (-41%)	-0.7 (-47%)	-1.7 (-58%)	-0.06 (-1%)	-0.08 (-5%)	0.03 (2%)	-0.05 (-5%)	-0.01 (-5%)				
	Mobility									-1.32 (-9%)	-0.04 (-24%)	0.02 (-)	0.01 (2%)

*Regional energy balance from 2016
** Regional energy balance from 2017
Entries of "-" for relative deviations imply that the comparison value was 0.

References for Supplementary Material

1. Länderarbeitskreis Energiebilanzen (LAK). *Energy Balance for the Federal State of North Rhine-Westphalia 2016*. (2021).
2. Länderarbeitskreis Energiebilanzen (LAK). *Energy Balance for the Federal State of Berlin 2017*. (2021).
3. Länderarbeitskreis Energiebilanzen (LAK). *Energy Balance for the Federal State of Mecklenburg-Western Pomerania 2016*. (2021).
4. Länderarbeitskreis Energiebilanzen (LAK). *Energy Balance for the Federal State of Saxony 2016*. (2021).
